# Supplementary material for: Identification of photoperiod-induced specific miRNAs in the adrenal glands of Sunite sheep (Ovis aries)
Source: Front Vet Sci. 2022 Jul 22;9:888207. doi: 10.3389/fvets.2022.888207 (PMC9354845; doi:10.3389/fvets.2022.888207)
Supplement: Supplementary file 1 [file Data_Sheet_1.ZIP › Supplementary file/miRDeep2 file.zip!/miRDeep2 file/Know_Structure/SP42A2/oar-let-7b.pdf]

miRBase precursor : oar-let-7b
Total read count : 211295
oar-let-7b read count : 210076
remaining reads : 1219

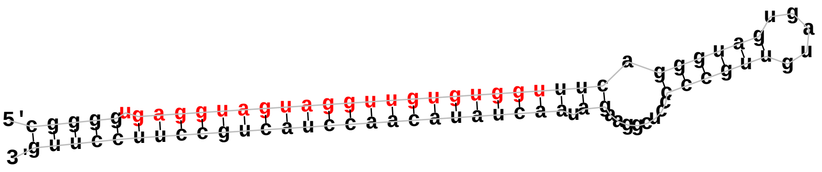

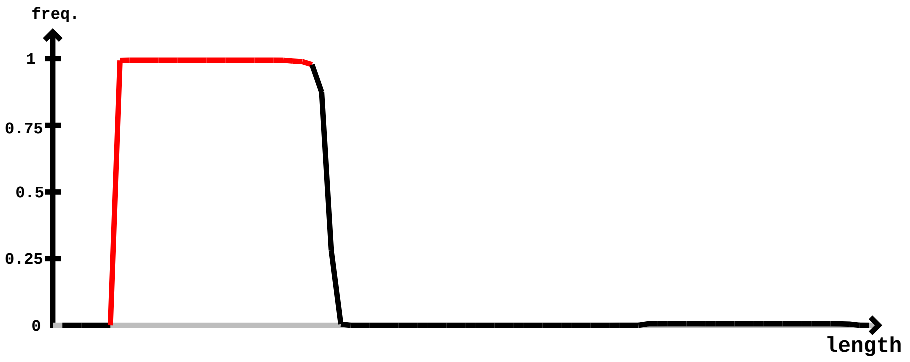

oar-let-7b

```
5'- cggggugagguaguagguuguguggguucaggguagugauguugcccccucggaagauaacuauacaaccuacugccuuccuug   -3'    exp
    (((((.(((((((((((((((((((((((.((((((.....))))))(....)..)).))))))))))))))))))))))))))    reads      mm         sample
    ....gugagguaguagguugugu.............................................................    1          0          seq
    ....gugagguaguagguugugugguuu........................................................    1          0          seq
    .....ugagguaguagguugug..............................................................    57         0          seq
    .....ugaCguaguagguugug..............................................................    1          1          seq
    .....ugagguaguaAguugug..............................................................    1          1          seq
    .....ugagguaguagguugugu.............................................................    639        0          seq
    .....ugagguaguaAguugugu.............................................................    1          1          seq
    .....ugagguaguagguugugug............................................................    462        0          seq
    .....ugagguaguagguuguguggg...........................................................    2378       0          seq
    .....ugagguaguagguugugugguu..........................................................    21747      0          seq
    .....ugagguaguagguugugugguu.........................................................    125136     0          seq
    .....ugagguaguagguugugugguuu........................................................    58591      0          seq
    .....ugagguaguagguugugugguuuc.......................................................    877        0          seq
    .....ugagguaguagguugugugguuuca......................................................    4          0          seq
    ......gagguaguagguugugu.............................................................    1          0          seq
    ......gagguaguagguugugugg...........................................................    1          0          seq
    ......gagguaguagguuguguggu..........................................................    25         0          seq
    ......gagguaguagguugugugguu.........................................................    93         0          seq
    ......gagguaguagguugugugguuu........................................................    50         0          seq
    .......agguaguagguuguguggu..........................................................    1          0          seq
    .......agguaguagguugugugguu.........................................................    4          0          seq
    ........gguaguagguugugugguu.........................................................    1          0          seq
    ........gguaguagguugugugguuu........................................................    1          0          seq
    ..........uaguagguugugugguu.........................................................    1          0          seq
    ..........uaguagguugugugguuu........................................................    2          0          seq
    ...........................ucaggguagugauguugc.......................................    1          0          seq
    ...........................ucaggguagugauguugcccccucgga..............................    1          0          seq
    ...........................ucaggguagugauguugcccccucggaa.............................    1          0          seq
    ............................................cccccucggaagauaacuaua...................    1          0          seq
    ............................................cccccucggaagauaacuauac..................    1          0          seq
    .............................................ccccucggaagauaacuauac..................    1          0          seq
    ..............................................cccucggaagauaacuauacaac...............    1          0          seq
    .....................................................aagauaacuauacaaccuacugccu......    2          0          seq
    ......................................................agauaacuauacaaccuacugc........    2          0          seq
    ........................................................auaacuauacaaccuacugccuu.....    1          0          seq
```

oar-let-7b

cggggugagguaguagguugugugguuucaggguagugauguugcccccucggaagauaacuauacaaccuacugccuuccuug			
....auaacuauacaaccuacugccuuccu..	1	0	seq
....uaacuauacaaccuacugcc.......	1	0	seq
....uaacuauacaaccuacugccuuc....	3	0	seq
....uaacuauacaaccuacugccuucc...	1	0	seq
....aacuauacaaccuacugccuu.....	1	0	seq
....aacuauacaaccuacugccuuc....	1	0	seq
....aacuauacaaccuacugccuucc...	1	0	seq
....cuauacaaccuacugccuu.....	3	0	seq
....cuauacaaccuacugccuuc....	29	0	seq
....cuauacaaccuacugccuucc...	233	0	seq
....cuauacaaccuacugccuuccu..	882	0	seq
....cuauacaaccuacugccuuccuu.	44	0	seq
....cuauacaaccuacugccuuccuug	1	0	seq
....uauacaaccuacugccuuc....	1	0	seq
....uauacaaccuacugccuuccu..	1	0	seq
....uauacaaccuacugccuuccuu.	1	0	seq
....auacaaccuacugccuucc...	1	0	seq
....auacaaccuacugccuuccu..	2	0	seq
